# Supplementary figures and images for: ZmIAA5 regulates maize root growth and development by interacting with ZmARF5 under the specific binding of ZmTCP15/16/17
Source: PeerJ. 2022 Jul 14;10:e13710. doi: 10.7717/peerj.13710 (PMC9288822; doi:10.7717/peerj.13710)

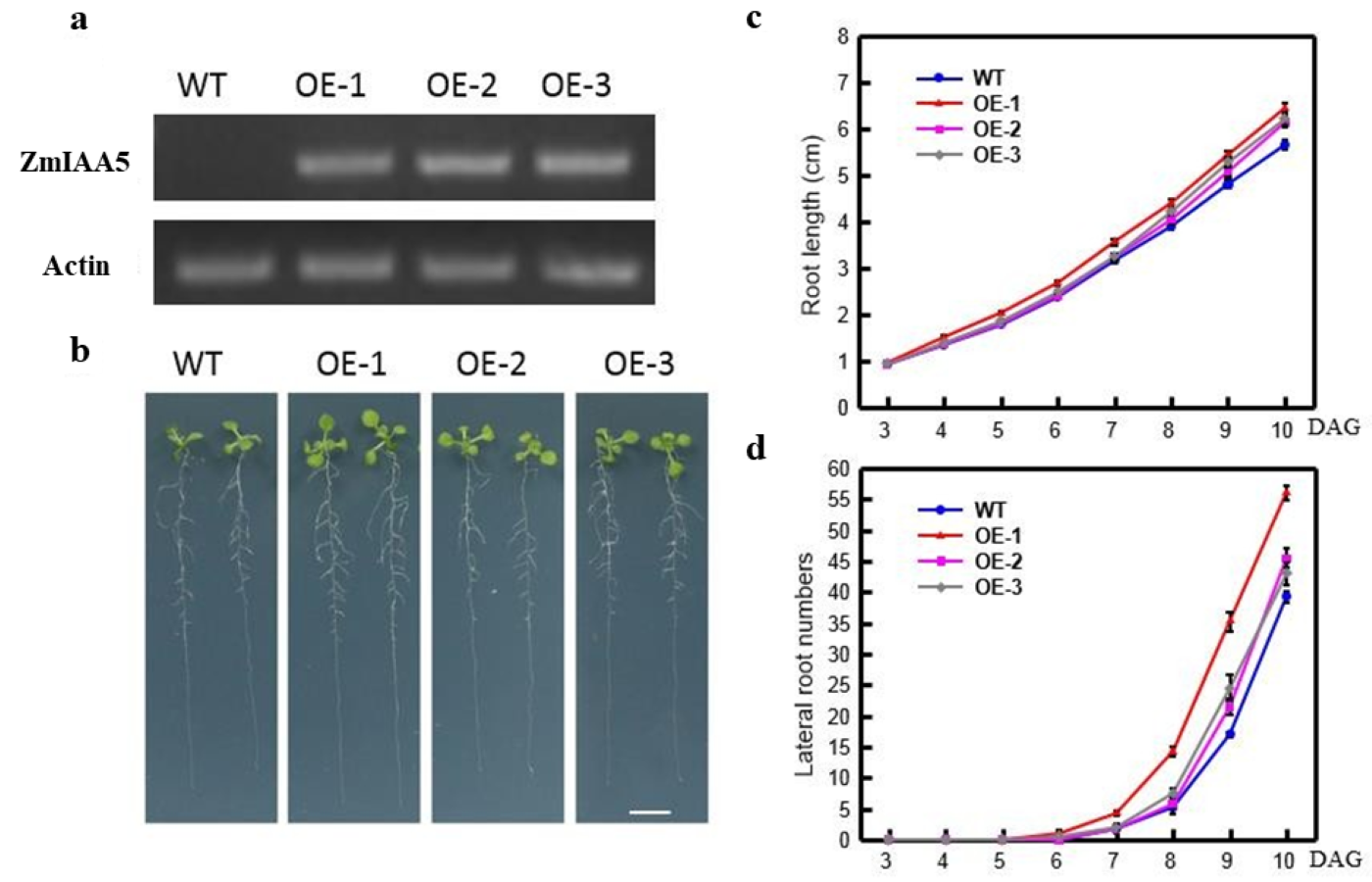

Supplement: Figure S1 — (a) Results of semi-quantitative analysis of ZmIAA5 gene. Three ZmIAA5 overexpressing lines were identified by semi-quantitative analysis. (b)Comparison of root phenotypes between overexpressed and wild-type plants. The overexpressed plants had longer main root length and more lateral roots than wild type. (10 days in MS medium) (c) Statistical chart of main root length change. Overexpressed plants had longer taproot lengths than wild type (from day 3 to day 10 of MS medium culture) (d) Statistical chart of changes in the number of lateral roots. Overexpression plants had more lateral roots than wild type (from day 3 to day 10 in MS medium). [file peerj-10-13710-s001.png]

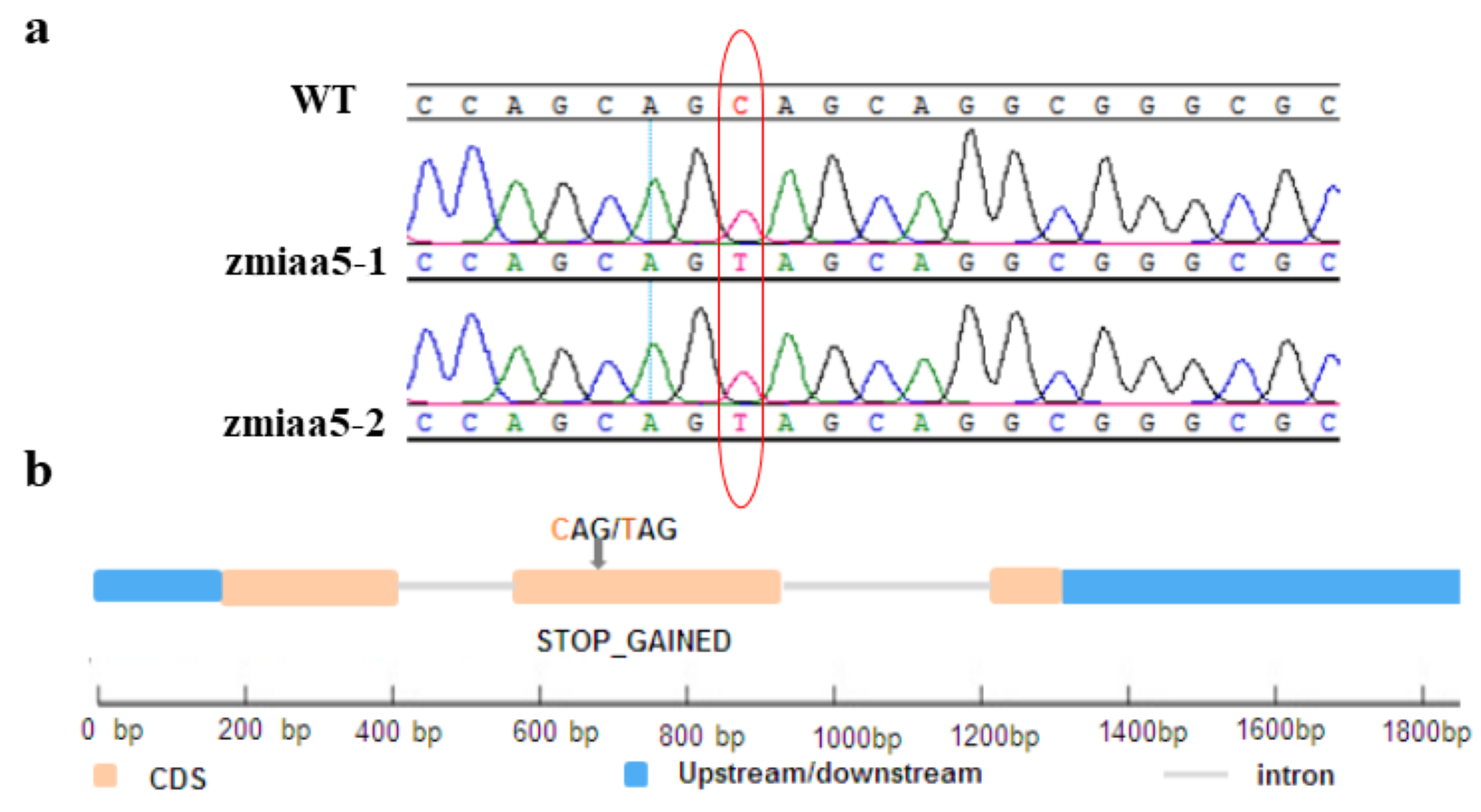

Supplement: Figure S2 — (a) Sequencing results of base mutation sites in maize zmiaa5 mutants. The zmiaa5-1 and zmiaa5-2 mutants are both the base C at a specific position mutated into T, resulting in the termination of transcription. (b)Schematic diagram of the mutation site of the zmiaa5 mutant. The mutation site of the two strains is located at 623bp of the genome of ZmIAA5. [file peerj-10-13710-s002.png]

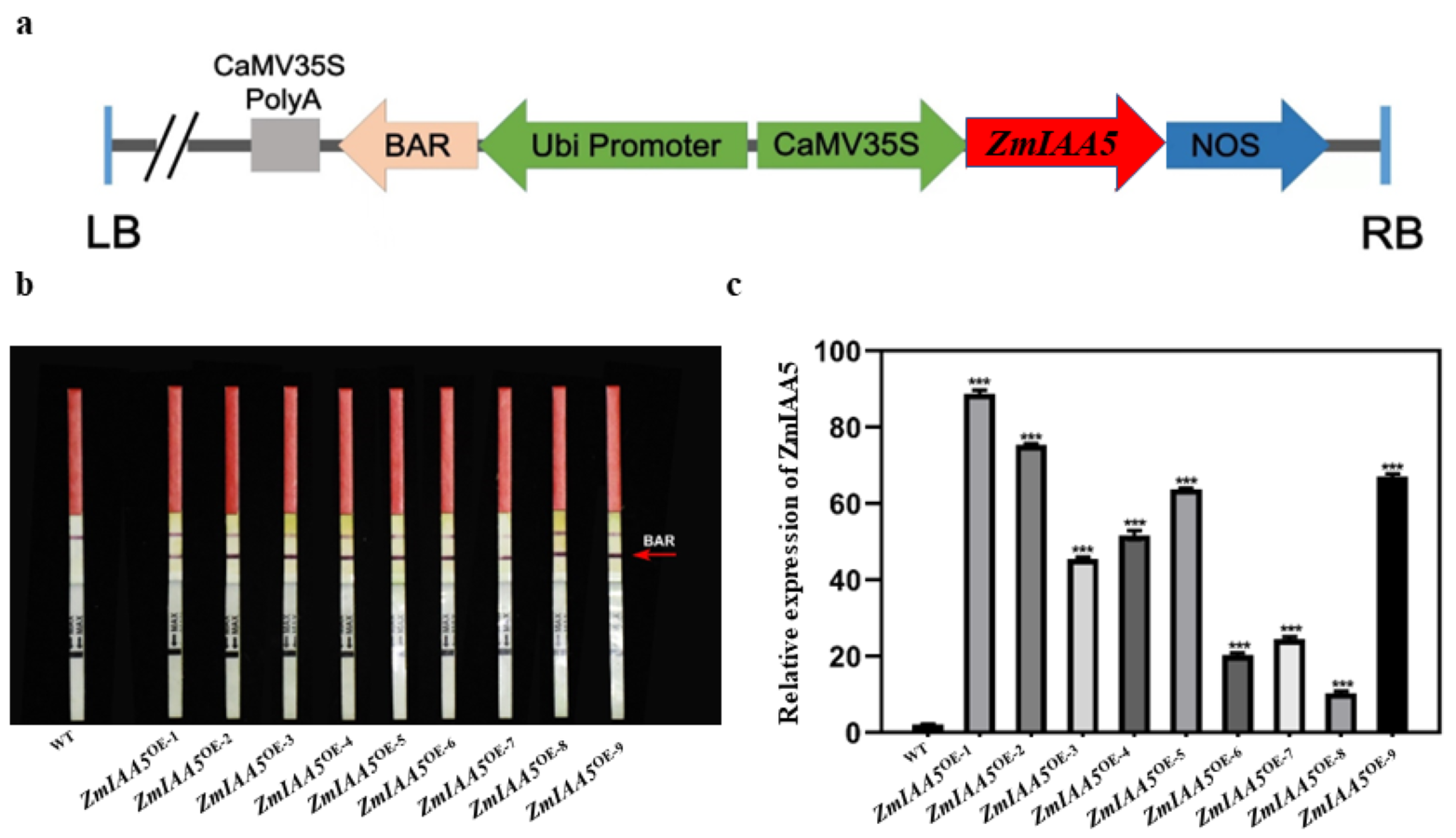

Supplement: Figure S3 — (a) Schematic diagram of constructing ZmIAA5 overexpression vector. (b) Positive plants detected by Bar test strip method. Two red bands on the Bar test strip indicate that the plant is positive. (c) Plants with positive gene expression detection. The ZmIAA5 gene was overexpressed in all nine lines. [file peerj-10-13710-s003.png]

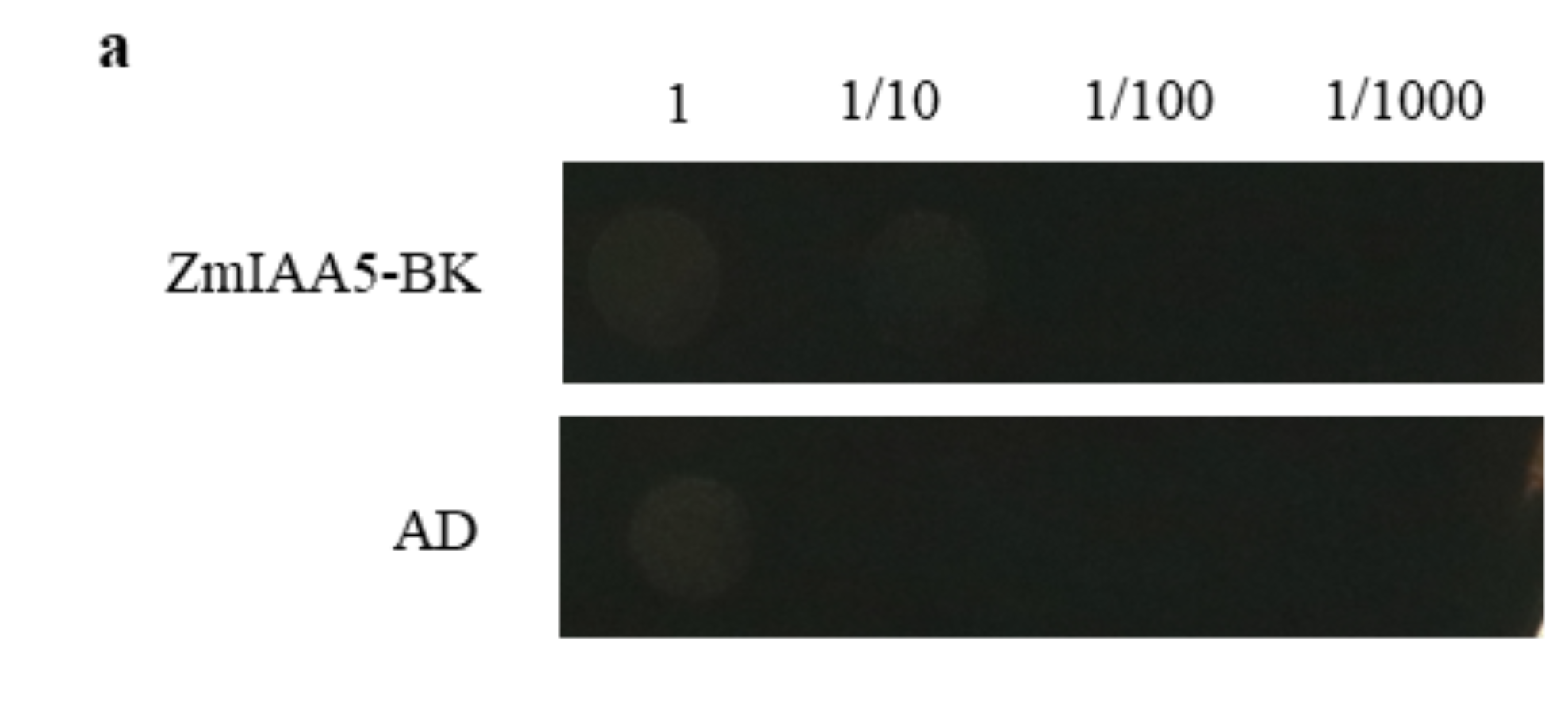

Supplement: Figure S4 — (a) Yeast two-hybrid experiment verified the self-activation of ZmIAA5 gene transcription. Plaques did not grow on the plate, proving that the ZmIAA5 gene has no transcriptional autoactivation. [file peerj-10-13710-s004.png]
